# Low-Velocity Impact Analysis in Composite Plates Incorporating Experimental Interlaminar Fracture Toughness

**DOI:** 10.3390/ma17235768

**Published:** 2024-11-25

**Authors:** Gyeong-Han Lee, Ji-Yoon Yang, Sang-Woo Kim, Soo-Yong Lee

**Affiliations:** 1 Department of Aerospace and Mechanical Engineering, Korea Aerospace University, Goyang-si 10540, Gyeonggi-do, Republic of Korea; ghanlee94@kau.kr; 2 Research Institute for Aerospace Engineering and Technology, Korea Aerospace University, Goyang-si 10540, Gyeonggi-do, Republic of Korea; leesy@kau.ac.kr; 3 Department of Cargo Containment System Research, Hyundai Heavy Industry, Seongnam-si 13553, Gyeonggi-do, Republic of Korea; jiyoon819@hd.com; 4 Department of Aeronautical and Astronautical Engineering, Korea Aerospace University, Goyang-si 10540, Gyeonggi-do, Republic of Korea

**Keywords:** Taguchi design, double cantilever beam (DCB) test, end-notched flexure (ENF) test, interlaminar fracture toughness, low-velocity impact, impact simulation

## Abstract

Reliable performance of composite adhesive joints under low-velocity impact is essential for ensuring the structural durability of composite materials in demanding applications. To address this, the study examines the effects of temperature, surface treatment techniques, and bonding processes on interlaminar fracture toughness, aiming to identify optimal conditions that enhance impact resistance. A Taguchi experimental design and analysis of variance (ANOVA) were used to analyze these factors, and experimentally derived toughness values were applied to low-velocity impact simulations to assess delamination behavior. Sanding and co-bonding were identified as the most effective methods for improving fracture toughness. Under the identified optimal conditions, the low-velocity impact analysis showed a delamination area of 319.0 mm^2^. These findings highlight the importance of parameter optimization in enhancing the structural reliability of composite adhesive joints and provide valuable insights for improving the performance and durability of composite materials, particularly in aerospace and automotive applications.

## 1. Introduction

Composite materials are defined as materials composed of two or more constituents, typically reinforced fibers and a matrix. These fiber-reinforced composites, known for their lightweight and superior strength-to-weight ratio, find applications in aerospace, automotive, and sports equipment [1]. However, due to their anisotropic nature and interlaminar interfaces, composites are vulnerable to impact loading [2,3,4]. In particular, interlaminar delamination caused by internal cracks in composites can degrade structural performance and potentially lead to structural failure. Interlaminar delamination in composites occurs readily due to interlayer or material property mismatches, particularly under impact loading [5]. Therefore, when low-velocity impact occurs on composite structures, it is crucial to consider not only intralaminar damage but also interlaminar delamination. To address this, understanding the interlaminar fracture toughness, which indicates the resistance to crack propagation, is essential.

The factors influencing interlaminar fracture toughness include temperature, surface treatment techniques, and bonding processes. These factors were chosen for their practical importance in determining the delamination resistance of composite adhesive joints. Temperature is critical as it affects the mechanical properties of the adhesive and its interaction with composite adherends across a range of environmental conditions. Surface treatment techniques, such as peel ply, fuse plasma, and sanding, were selected due to their widespread use in improving adhesive strength and interlaminar fracture toughness. Bonding processes, including co-bonding and secondary bonding, were chosen to evaluate the distinct effects on the mechanical behavior of composite joints. Sales et al. [6] aimed to investigate the effect of temperature on mode I and mode II interlaminar fracture toughness through Double Cantilever Beam (DCB) and End-Notched Flexure (ENF) tests. Hashemi et al. [7] derived mode I, mode II, and mixed-mode interlaminar fracture toughness across a temperature range from 20 °C to 130 °C, while Sales et al. [8] reported results at specific temperatures of 54 °C, 25 °C, and 80 °C. These findings confirmed an increase in interlaminar fracture toughness with increasing temperature.

Various surface treatment techniques aim to enhance the adhesive strength and interlaminar fracture toughness at the interface between composites and adhesives. These methods include peel ply treatment, sanding, and fuse plasma treatment. Albertsen et al. [9] conducted mode I, mode II, and mixed-mode tests, confirming that surface treatments increase interlaminar fracture toughness. Aliheidari et al. [10] compared mode II interlaminar fracture toughness among untreated specimens, sanded specimens, and plasma-treated specimens, finding higher toughness in the surface-treated groups. Similarly, Martínez-Landeros et al. [11] investigated mode I interlaminar fracture toughness in composite adhesive joints after treatments such as solvent cleaning, sanding, chemical etching, and peel ply treatment, and analyzed the damage modes of the joints. In addition to surface treatment factor, Blythe et al. [5] demonstrated that polyamide nanofiber veils in hybrid carbon/glass composites effectively localized cracks and reduced delamination, enhancing both fracture toughness and pseudo-ductility under quasi-static loads.

**Figure 1:**
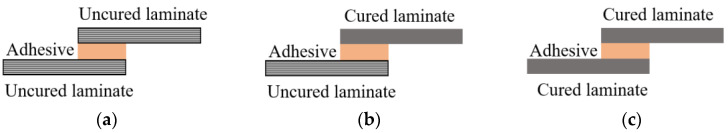
Adhesive bonding of composite materials: (**a**) co-curing; (**b**) co-bonding; (**c**) secondary bonding.

In the fabrication of composite structures, mechanical fastening with bolts and nuts and adhesive bonding using various adhesion processes are commonly employed. Adhesion processes for laminating composite panels, as illustrated in Figure 1, include co-curing, co-bonding, and secondary bonding [12]. The co-curing process is a method where the adhesive layer between the two uncured laminates is also cured simultaneously, as shown in Figure 1a. The co-bonding process consists of curing the adhesive between a pre-cured laminate and an uncured laminate, with the adhesive layer curing simultaneously with the uncured laminate, as shown in Figure 1b. Meanwhile, the secondary bonding process involves curing the adhesive between pre-cured laminates, as illustrated in Figure 1c. Brito et al. [13] compared the mode I interlaminar fracture toughness of joints created using simultaneous and secondary bonding techniques, discovering that simultaneous bonding resulted in higher toughness at the adhesive joint where cracks occurred. Similarly, Shiino et al. [14] found that joints fabricated using simultaneous bonding exhibited higher mode I interlaminar fracture toughness compared to those made with resin infusion. These findings demonstrate that interlaminar fracture toughness varies significantly depending on the adhesion process used.

This study investigates the influence of temperature, surface treatment techniques, and adhesion processes on mode I and mode II interlaminar fracture toughness of composite adhesive joints through experimental validation. Experimental designs were developed using the Taguchi method, followed by analysis of variance to assess their impact. Additionally, the interlaminar fracture toughness obtained from experiments was applied to conduct low-velocity impact analysis of composite panels, comparing damage areas under different conditions of composite adhesive joints. Finally, optimal conditions for enhancing interlaminar fracture toughness are proposed.

## 2. Experiments for Interlaminar Fracture Toughness

### 2.1. Taguchi Design of Experiments

The Taguchi method simplifies experimental design by using advanced statistical analysis of input variables to achieve optimal results, thereby improving both efficiency and quality. This method has been developed to design experiments using orthogonal arrays to study how the variance and mean of a process are affected by different parameters [15,16]. In this process, the focus is on analyzing the main effects and interactions to derive optimal conditions.

The main effect denotes the average influence of individual factors on test outcomes. In the main effects plot, lines that approach horizontality indicate minimal influence, while steeper slopes signify a greater effect of the corresponding factor. Thus, the main effects plot is crucial in identifying significant factors. Interaction effects describe situations where the impact of one factor depends on the levels of another factor, requiring interaction analysis when multiple factors are involved. Parallel lines in the interaction plot signify no interaction, whereas increasing slope differences between lines indicate a higher degree of interaction. Therefore, the Taguchi method was employed to analyze the effects of temperature, surface treatment, and bonding methods on the interlaminar fracture toughness of composites. Response factors and experimental factors were selected for both tests, along with the levels of each experimental factor, as detailed in Table 1.

The factors for the DCB test are designated as A, B, and C. Factor A represents the temperature, which includes low temperature (−53 °C, CTA), room temperature (23 °C, RTA), and high temperature (93 °C, ETA). Factor B signifies the surface treatment methods, including peel ply (PP) treatment, fuse plasma (FP) treatment, and sanding (SD). Factor C refers to the bonding processes, which include co-bonding (CB) and secondary bonding (SB). Peel ply treatment involves attaching and removing a textured fabric to the surface of the prepreg before curing. Sanding is the method of abrading the surface using sandpaper or other abrasive tools. Fuse plasma treatment uses high-energy gas to remove surface contaminants and chemically activate the surface to enhance adhesive performance. To design the DCB tests with the minimum number of runs, a mixed-level design was implemented, resulting in an L18 orthogonal. Based on this orthogonal array, five valid data points were obtained for each experimental condition.

Meanwhile, the factors for the ENF test regarding surface treatment and the adhesion process are designated as D and E, respectively. Factor D represents the surface treatment methods, including PP, FP, and SD, while Factor E represents the bonding processes, including CB and SB. Similar to the DCB test, a mixed-level design was employed, generating an L6 orthogonal. Five valid data points were collected for each experimental condition.

### 2.2. Double Cantilever Beam and End-Notched Flexure Tests

**Figure 2:**
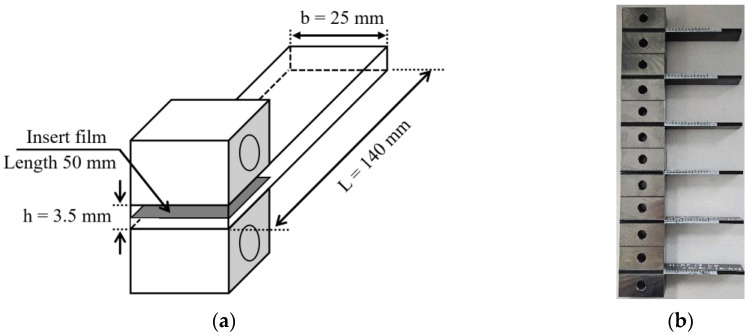
DCB specimen and its dimension: (**a**) dimension of the DCB specimen; (**b**) samples.

**Figure 3:**
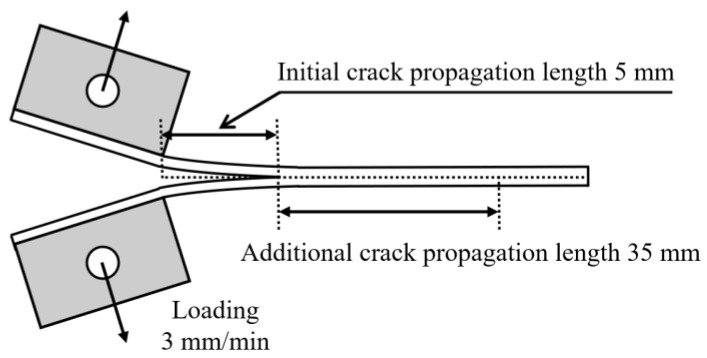
Schematic diagram of DCB tests.

**Figure 4:**
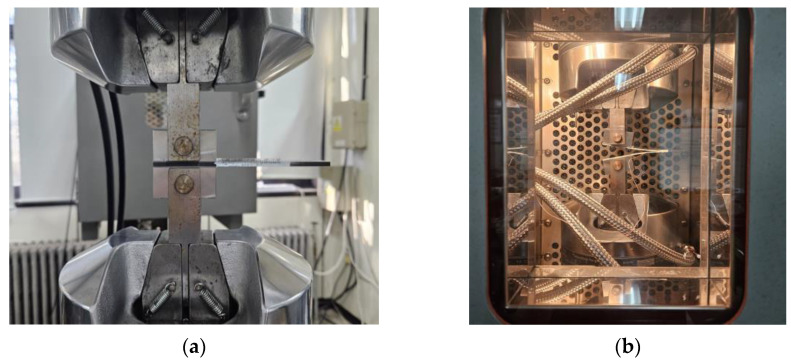
Photos of mounting of DCB specimens for DCB tests: (**a**) at room temperature; (**b**) in environmental test oven.

To determine the mode I interlaminar fracture toughness at the adhesive interface of composites, DCB tests were conducted based on ASTM D5528 [17] standards. The geometry of the DCB specimen is presented in Figure 2, and its dimensions are given as a length of 140 mm, a width of 25 mm, and a thickness of 3.5 mm, as shown in Figure 2a. A Teflon film, which is a non-adhesive film, was inserted according to the insert length of the specimen. Tensile loads were applied by attaching metal loading blocks on the upper and lower sides of the specimen. Preliminary tests induced an initial crack growth of approximately 5 mm. To enable the observation of crack propagation, a white marker was used, as shown in Figure 2b. After removing the load, the test was conducted again, resulting in an additional crack growth of over 35 mm. The test speed was maintained at 3 mm/min. The schematic of the test is shown in Figure 3. Figure 4 shows the photos of mounting of DCB specimens for the DCB tests. An MTS-810 universal testing machine, manufactured by MTS Systems Corporation, USA, was used to perform the DCB test. For low and high-temperature environments, an MTS 651 environmental chamber for structural testing was utilized. The OMEGA DP-41 thermocouple, manufactured by OMEGA Engineering, Norwalk, USA, was used to measure the test environment and specimen temperatures. The DCB test conducted at room temperature is presented in Figure 4a, and the photograph of the specimen mounted in the environmental chamber is shown in Figure 4b.

**Figure 5:**
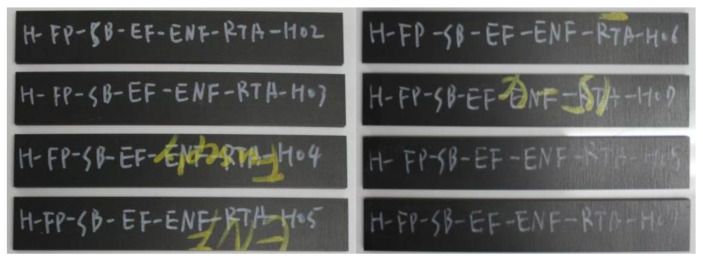
Samples of ENF specimen.

**Figure 6:**
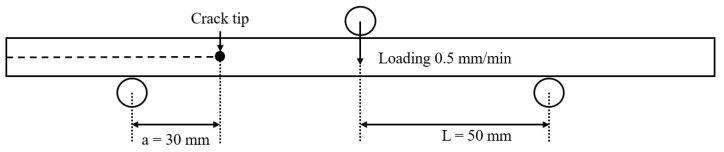
Schematic diagram of ENF tests.

The mode II interlaminar fracture toughness tests were conducted in accordance with ASTM D7905 [18] standards. The ENF specimen geometry is identical to that of the DCB specimen, but the loading blocks were removed since bending loads were applied as shown in Figure 5. Figure 6 shows the schematic of the ENF test, with a span length of 100 mm. The test was conducted until the crack tip reached 30 mm from the left support, at the test speed of 0.5 mm/min.

**Figure 7:**
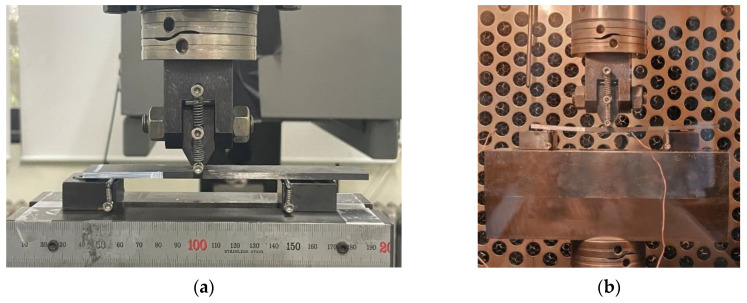
Photos of mounting of ENF test specimens: (**a**) at room temperature; (**b**) in environmental test oven.

Figure 7 depicts the test specimen mounted on the universal testing machine. Figure 7a shows the specimen at room temperature. In accordance with the DCB test, low- and high-temperature tests were conducted using an environmental chamber, manufactured by MTS Systems Corporation, USA, as shown in Figure 7b. The ENF test was first performed as a non-precracked (NPC) test to calculate the crack length using the following equation:

(1) $$a_{calc}={\left(\frac{C_{u}-A}{m}\right)}^{\frac{1}{3}},$$

(2) $$C_{u}=A+ma^{3},$$

where *C_u_* (mm/N) is the compliance of the unloading line, and *A* (mm/N) and *m* (1/N·mm^−2^) are the correlation coefficients from the NPC test to be determined using a linear least squares regression analysis of the compliance *C_u_*. More specifically, *A* is the intercept and m is the slope obtained from the regression analysis. Subsequently, a pre-cracked (PC) test is performed with the newly applied crack length, and mode II interlaminar fracture toughness is calculated using the following equation:

(3) $$G_{PC}=\frac{3mP_{max}^{2}a_{PC}^{2}}{2B},$$

where *G_PC_* (N/mm) is the mode II interlaminar fracture toughness value from the PC test, *m* is the correlation coefficient, *P*max is the maximum load from the PC fracture test, $a_{pc}$ is the crack length during the PC fracture test, and *B* is the specimen width.

### 2.3. Analysis of Variance for Interlaminar Fracture Toughness Tests

To analyze the significance of the experimental factors on the interlaminar fracture toughness test, an analysis of variance (ANOVA) was performed. ANOVA is a statistical technique that uses variance to compare the means of three or more groups. It expresses the dispersion of the characteristic values as the sum of squares and decomposes it into the sum of squares for each experimental factor to identify the factors that have a significant impact. The ANOVA is performed using the following equations:

(4) DF=k−1,

(5) $$SS=\frac{1}{n}\sum_{i=1}^{L}{\left(Y_{i}-\bar{Y}\right)}^{2},$$

(6) MS=SS/DF,

(7) $$F=MS_{A}/MS_{E},$$

here, *DF* denotes the degrees of freedom for the factor, *SS* represents the sum of squares of the differences between each observation and the mean, n is the number of repetitions, *k* is the number of levels of the factor, Y is the observation value of the factor, and $\bar{Y}$ is the mean of the observations. *MS* is the value obtained by dividing the sum of squares by the corresponding degrees of freedom. *F* indicates how much the variation of each factor is greater than the error variation and is calculated by dividing the mean square of the factor by the mean square of the error. The subscripts *A* and *E* denote the factor and the error, respectively. Based on the *F* value, the *p*-value is derived, which indicates the contribution of the experimental factor to the response variable.

## 3. Low-Velocity Impact Simulation for Composite Plate

### 3.1. Damage Model for Composite Plate

#### 3.1.1. Intralaminar Damage Model

Composite materials are manufactured by combining two or more distinct materials, such as anisotropic fibers and isotropic resins [19]. The damage mechanisms of orthotropic composite materials differ from those of isotropic materials and are more complex. Therefore, an appropriate failure model must be applied to predict the initiation and evolution of damage in composites. A representative damage criterion is the Hashin theory, which presents four criteria for damage initiation [20]. The four composite damage modes (fiber tension, fiber compression, matrix tension, and matrix compression) are identified using the Hashin damage initiation criteria, as shown in the following equations [21,22]:

(8) $$F_{f}^{t}={\left(\frac{\sigma_{11}}{X^{T}}\right)}^{2}+\alpha {\left(\frac{\tau_{12}}{S^{L}}\right)}^{2},$$

(9) $$F_{f}^{c}={\left(\frac{\sigma_{11}}{X^{C}}\right)}^{2},$$

(10) $$F_{m}^{t}={\left(\frac{\sigma_{22}}{Y^{T}}\right)}^{2}+{\left(\frac{\tau_{12}}{S^{L}}\right)}^{2},$$

(11) $$F_{m}^{c}={\left(\frac{\sigma_{22}}{2S^{T}}\right)}^{2}+\left[{\left(\frac{Y^{C}}{2S^{T}}\right)}^{2}-1\right]\frac{\sigma_{22}}{Y^{C}}+{\left(\frac{\tau_{12}}{S^{L}}\right)}^{2},$$

here, *X^T^* is the tensile strength in the fiber direction, *X^C^* is the compressive strength in the fiber direction, *Y^T^* is the tensile strength perpendicular to the fiber direction, *Y^C^* is the compressive strength perpendicular to the fiber direction, *S^L^* is the longitudinal shear strength, *S^T^* is the transverse shear strength, *α* is the coefficient determining the contribution of shear stress in the fiber tension criterion, and *σ* and *τ* are the components of the effective stress tensor. The damage evolution of composites in the Hashin theory can be defined through the variables *G_ft_*, *G_fc_*, *G_mt_*, and *G_mc_*, which represent the energies dissipated during damage for fiber tension, fiber compression, matrix tension, and matrix compression failure modes, respectively [22].

#### 3.1.2. Interlaminar Damage Model

**Figure 8:**
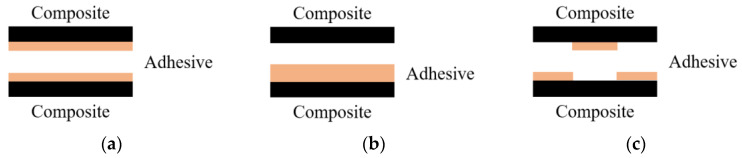
Damage mode shape of adhesive joints: (**a**) cohesive failure mode; (**b**) adhesive failure mode; (**c**) mixed failure mode.

In general, because the properties of adhesives are weaker than those of composites, damage occurs at the bonded joints of composites. The types of damage that can occur at the bonded joints are shown in Figure 8 [23]. Figure 8a illustrates the cohesive failure mode, where cracks occur within the adhesive. Figure 8b shows the adhesive failure mode, where damage occurs at the interface between the composite and the adhesive, and Figure 8c depicts the mixed failure mode, where both types of damage occur simultaneously.

A prominent analytical approach for predicting progressive damage within bonded joints of composites includes CZM (cohesive zone model), VCCT (virtual crack closure technique), and XFEM (extended finite element method) [24]. CZM defines the initiation and propagation of cracks using parameters such as adhesive strength and damage energy. In contrast, VCCT predicts crack propagation direction similar to CZM but does not account for initial damage. XFEM is useful for predicting complex crack shapes by representing cracks within elements of a localized model. In this study, CZM, which can simulate both the formation of initial cracks and their progression, was applied to model the bonded joints.

**Figure 9:**
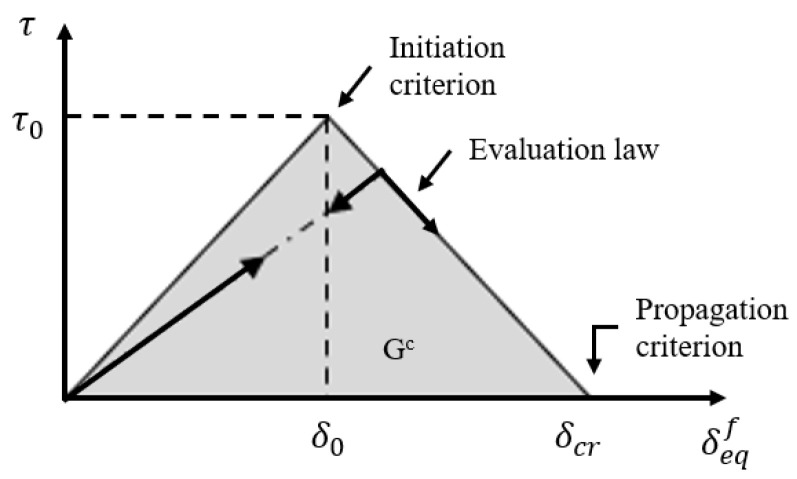
The bilinear traction–separation graph.

CZM is a technique that defines properties for initial damage and progressive crack growth to facilitate modeling. It defines the stress in the interfacial separation zone using a traction–separation law. Examples of traction–separation laws include bilinear, exponential, and parabolic forms [25]. Alfano et al. [26] confirmed through their research that there is little difference between traction–separation relationships when modeling adhesive regions and simulating interfacial separation. Therefore, this study employs a relatively simple bilinear traction–separation law to model the adhesive. The bilinear traction–separation law can be represented as a function of stress (traction) in the damaged area and crack opening displacement. As shown in the traction–separation graph in Figure 9, the load increases linearly just before the initiation of initial cracks. The slope K of this graph indicates stiffness [27]. After initial damage, the load decreases linearly, and the area under the graph represents the damage energy G^c^ [28]. Therefore, to simulate interfacial separation in analysis, it is necessary to apply the traction and damage energy of the materials used in the CZM area.

There are various damage assessment criteria for CZM elements, including the maximum nominal stress criterion (MAXS damage) and the quadratic nominal stress criterion (QUADS damage). Da Rocha et al. [29] conducted research indicating that the QUADS damage criterion offers higher predictive accuracy among different damage assessment criteria. Therefore, this study aims to predict initial damage and progressive damage using the QUADS damage criterion, expressed by the following equation:

(12) $${\left(\frac{\langle t_{n}\rangle }{T_{n}}\right)}^{2}+{\left(\frac{\langle t_{s}\rangle }{T_{s}}\right)}^{2}+{\left(\frac{\langle t_{t}\rangle }{T_{t}}\right)}^{2}=1,$$

here, $t_{n}$, $t_{s}$, and $t_{t}$ represent the value of nominal stresses in normal, first shear, and second shear direction, respectively. $T_{n}$, $T_{s}$, and $T_{t}$ represent the maximum allowable nominal stresses in each direction.

Meanwhile, the loads applied to the bonded joints of composites involve mixed-mode loading of mode I, mode II, and mode III, necessitating consideration of their interrelation. For progressive damage assessment in this study, the Benzeggagh–Kenane criterion (B–K criterion), an energy-based damage assessment method, was applied. The B–K criterion is useful when the critical damage energies for mode I and mode II shear are equal (*G_s_^C^* = *G_t_^C^*). Assuming equal properties for mode I and mode II shear, the following equation representing the B–K criterion was applied in this study:

(13) $$G_{n}^{C}+\left(G_{s}^{C}-G_{n}^{C}\right){\left\{\frac{G_{S}}{G_{T}}\right\}}^{\eta }=G^{C},$$

here, *G^C^*, *G_n_^C^*, and *G_s_^C^* refer to the total, normal, and shear critical fracture energy, respectively. *G_S_ = G_s_ + G_t_*, *G_T_ = G_n_ + G_S_ + G_t_*, representing the dissipated energy in the out-of-plane direction and in all three directions, respectively. *η* is a cohesive property parameter typically set to 1.45 for carbon fiber composites.

### 3.2. Low-Velocity Impact Simulation for Composite Plate Considering Interlaminar Fracture Toughness

#### 3.2.1. Finite Element Model for Verification

**Figure 10:**
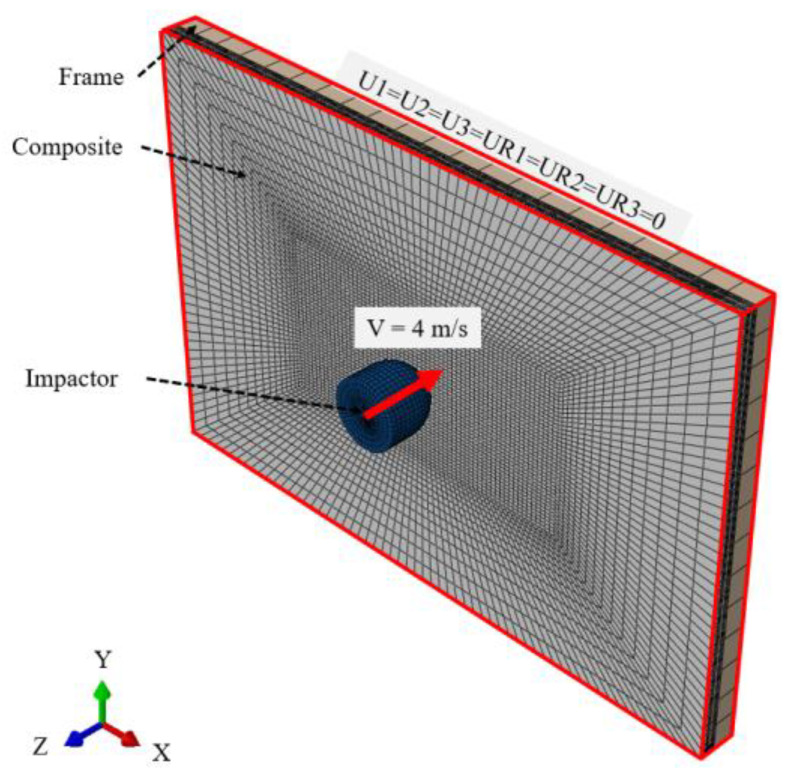
Finite element model of composite panel for impact analysis validation.

To validate the impact analysis for composite panels using CZM, comparisons were made with the results of Zhang et al. [30], and the model for a low-velocity impact analysis similar to Figure 10 was constructed. The carbon fiber composite panel measures 150 mm × 100 mm × 4 mm, with a stacking sequence of [0_2_, 45_2_, 90_2_, −45_2_]_s_. The applied composite material is T700/M21, and Table 2 and Table 3 present the material properties used in the analysis [30]. The impactor, defined as a 2 kg mass of steel, was modeled with a diameter of 16 mm and a total length of 15 mm. The frame was also defined as steel, with external dimensions of 150 mm × 100 mm × 5 mm and internal dimensions of 125 mm × 75 mm × 5 mm, featuring an open inner configuration. All four edges of the composite plate were constrained with six degrees of freedom (U1 = U2 = U3 = UR1 = UR2 = UR3 = 0).

Each layer of the composite panel was modeled using 3D continuum shell elements, employing 8-node SC8R elements. Cohesive zones were introduced between layers using 3D 8-node cohesive elements (COH3D8). The panel used 8-node hexahedral shell elements (SC8R), while the impactor and frame utilized 4-node quadrilateral solid elements (R3D4). Element sizes within the central area of the panel 72 mm × 36 mm were set at 1.2 mm × 0.9 mm × 0.5 mm, increasing gradually away from the center. Element sizes for the impactor and frame were 1.5 mm and 5 mm, respectively. The total number of elements and nodes in the entire model were 169,416 and 254,912, respectively.

The total analysis time was 4 ms, with each time step set to 0.02 ms. During the impact where penetration did not occur, general contact was applied throughout the entire model. Normal penetration was prevented, and a friction coefficient of 0.3 was applied in the tangential direction [30,31,32,33,34]. The impactor was fully constrained except in the initial step in the z-direction, with an initial velocity set to 5 m/s to achieve an initial impact energy of 25 J. The sides of the panel and the entire frame were constrained in all directions.

#### 3.2.2. Low-Velocity Impact Simulation Model

**Figure 11:**
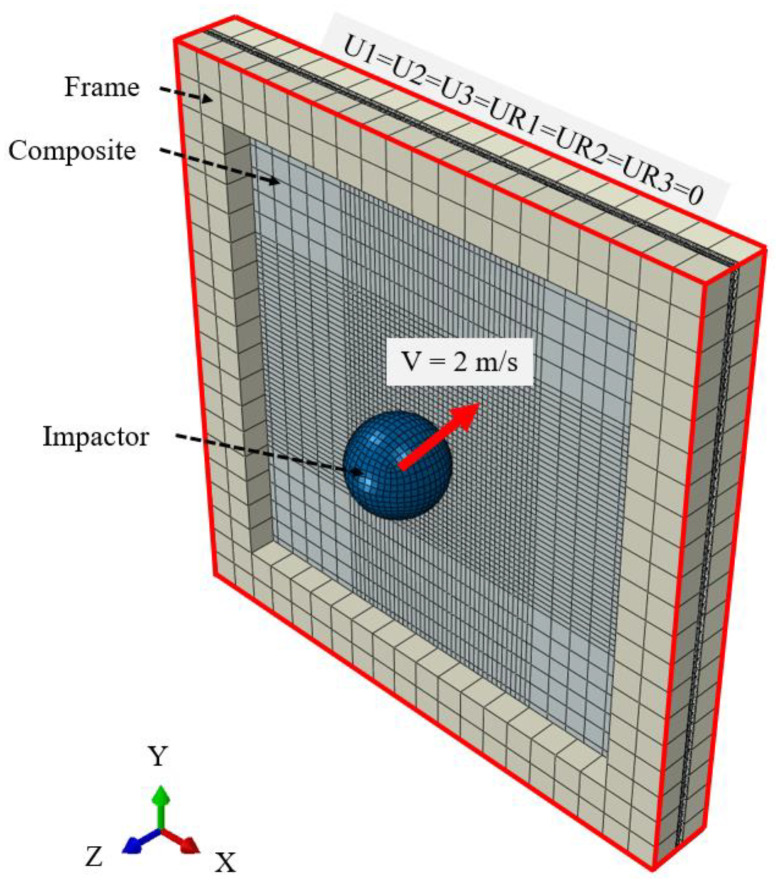
Finite element model of composite panel for low-velocity impact simulation.

We conducted low-velocity impact analysis of composite materials under various temperatures, surface treatments, and adhesion process conditions using ABAQUS/Explicit 2024. The impact analysis model consists of three parts, composite panel, impactor, and frame, as depicted in Figure 11. To account for intra-layer damage within the composite and interlayer separation at the adhesive interface, adhesive was additionally modeled between two composite laminates. Each composite laminate was a square of 100 mm × 100 mm with a total thickness of 1.6 mm. The composite is modeled with all layers oriented at 0°, and each layer had a thickness of 0.14 mm. The impactor was defined as a rigid body with a diameter of 16 mm and a mass of 1 kg. The frame, which secured the laminates front and back, is also defined as a rigid body, with external dimensions of 100 mm × 100 mm × 5 mm and internal dimensions of 80 mm × 80 mm × 5 mm, creating an open interior shape. All four edges of the frame were fully constrained by fixing all six degrees of freedom (U1 = U2 = U3 = UR1 = UR2 = UR3 = 0). The same materials used in the interlaminar fracture toughness tests were employed for the composite and adhesive. Thermal properties of the composite and adhesive were applied considering thermal expansion in the composite laminate due to temperature variations, as shown in Table 4 [35,36]. Table 5 and Table 6 present the mechanical properties used in the low-velocity impact analysis [37,38]. Table 7 provides the interlaminar fracture toughness of adhesive FM309. Damage in the laminates was assessed using the Hashin damage theory, while damage in the adhesive was analyzed using the QUADS damage theory during the impact analysis.

The composite laminate used the SC8R elements for the validation model, and the adhesive employed the cohesive element COH3D8. The impactor and frame were defined using rigid elements R3D4. The element size in the central region of the laminate 50 mm × 50 mm was set to 1 mm, increasing gradually away from the center. The element sizes for the impactor and frame were 1 mm and 5 mm, respectively. The total number of elements and nodes in the entire model were 43,716 and 87,630, respectively.

The total analysis time was set to 2 ms, with each time step set at 0.1 ms. General contact was applied throughout the entire model with a uniform friction coefficient of 0.3. The impactor was fully constrained in all directions except the z-direction from the initial step, and its velocity was set to 2 m/s to achieve an initial impact energy of 2 J. The edges of the laminate and the entire frame were constrained in all directions.

## 4. Results

### 4.1. Interlaminar Fracture Toughness Test

#### 4.1.1. Mode I

Table 8 presents the results of mode I interlaminar fracture toughness tests for composite adhesive specimens. Comparing the average values from the test results, specimens fabricated through the co-bonding adhesive process showed higher mode I interlaminar fracture toughness compared to those fabricated using the secondary adhesive process. This can be attributed to the chain interdiffusion mechanism in co-bonding, which facilitates chemical adhesion between the adhesive and the uncured laminate. In contrast, secondary bonding relies on mechanical interlocking, which provides relatively lower fracture toughness [39]. The differences in interlaminar fracture toughness due to surface treatment techniques revealed a decreasing trend in SD, PP, and FP treatments, respectively. Regarding temperature, the toughness decreased in the order of high, ambient, and low temperatures. Thus, an increase in temperature correlated with an increase in mode I interlaminar fracture toughness.

**Figure 12:**
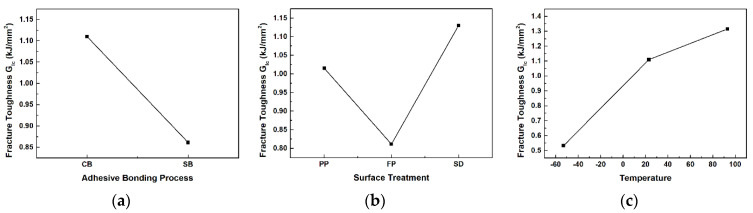
Main effect graphs for mean of mode I interlaminar fracture toughness: (**a**) adhesion process; (**b**) surface treatment; (**c**) temperature.

The response factor in Taguchi design is the mode I interlaminar fracture toughness, and it was analyzed based on three factors from DCB tests. The Taguchi design analysis results for the three factors in DCB tests are shown in Figure 12. Figure 12a depicts the main effects plot for the adhesion process factor on the average mode I interlaminar fracture toughness. Figure 12b illustrates the main effects plot for the surface treatment factor, and Figure 12c indicates the main effects plot for the temperature factor. As the main effects lines for all three factors are not horizontal, it indicates that each factor significantly influences the mode I interlaminar fracture toughness.

**Figure 13:**
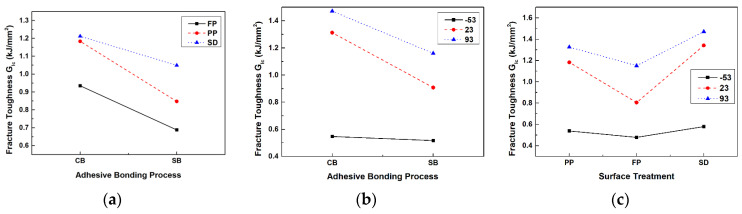
Interaction graphs for mean of mode I interlaminar fracture toughness: (**a**) adhesion process and surface treatment; (**b**) adhesion process and temperature; (**c**) surface treatment and temperature.

The interaction plot for the average mode I interlaminar fracture toughness is depicted in Figure 13. The interaction between the adhesion process and surface treatment is not parallel for all three lines, as shown in Figure 13a. This result indicates that the interaction between the adhesion process and surface treatment is significant. Figure 13b depicts the interaction plot between the adhesion process and test temperature. It was noted that the interaction lines for room temperature and high temperature are parallel to each other, whereas the line for low temperature is not parallel to them. Therefore, the interaction between the adhesion process and test temperature is significant at low temperatures. Figure 13c shows the interaction plot between surface treatment and test temperature. Since the interaction lines for each condition are not parallel, the interaction between surface treatment and temperature is significant.

To numerically verify the significance of main effects, the response factor selected was mode I interlaminar fracture toughness, and ANOVA was conducted. Table 9 presents the results of the ANOVA for mode I interlaminar fracture toughness. In the ANOVA results table, the significance probability (*p*-value) indicates whether each factor or interaction is statistically significant in relation to the response factor. If the *p*-value is less than or equal to the significance level (α), the association is statistically significant. Typically, using the significance level of 0.05, we examined the ANOVA results. The results show that the significance probabilities for all three factors, adhesion process, surface treatment, and temperature, are less than 0.05. This indicates that each factor has a statistically significant effect on the mode I interlaminar fracture toughness. Therefore, all three factors significantly influenced the mode I interlaminar fracture toughness.

#### 4.1.2. Mode II

Table 10 presents the results of mode II interlaminar fracture toughness tests on composite adhesive specimens. Similar to the mode I tests, it was observed that specimens fabricated using co-bonding adhesion processes exhibit higher mode II interlaminar fracture toughness compared to those using secondary adhesion processes. Additionally, differences in interlaminar fracture toughness were noted depending on the surface treatment techniques employed. Specifically, mode II interlaminar fracture toughness decreases in the following order: peel ply, fuse plasma, sanding.

**Figure 14:**
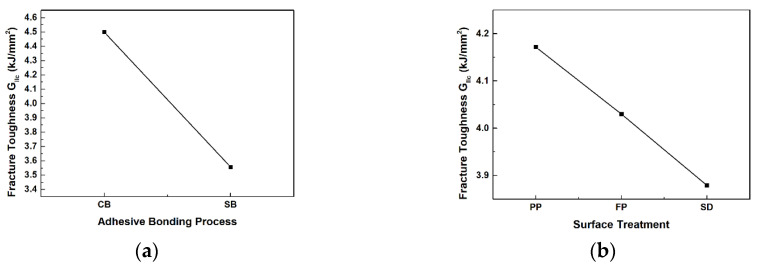
Main effect graphs for mean of mode II interlaminar fracture toughness: (**a**) adhesion process; (**b**) surface treatment.

The response factor in Taguchi design is the mode II interlaminar fracture toughness, and it was analyzed to study the effects of adhesion processes and surface treatments on mode II interlaminar fracture toughness. Figure 14 illustrates the main effects plot for the average mode II interlaminar fracture toughness based on adhesion processes and surface treatments. Figure 14a depicts the main effects plot for the adhesion process factor on the average of mode II interlaminar fracture toughness. Figure 14b illustrates the main effects plot for the surface treatment factor on the average of mode II interlaminar fracture toughness. As the main effects lines for the two factors are not parallel, it can be concluded that both factors have a significant impact on the mode II interlaminar fracture toughness.

**Figure 15:**
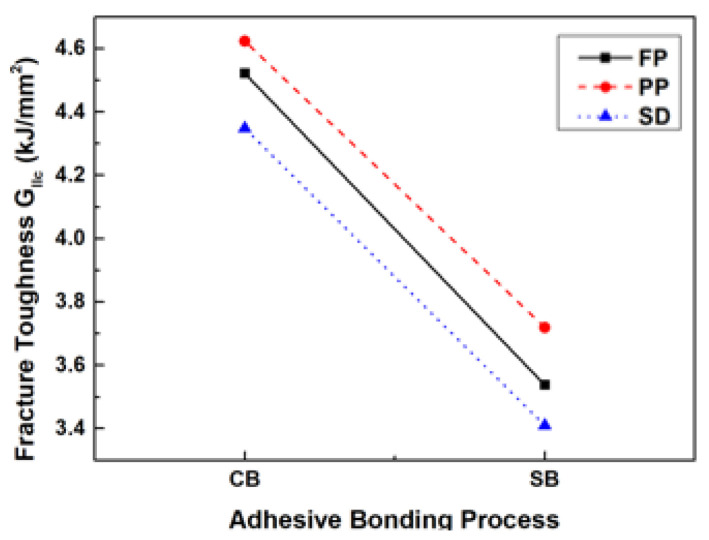
Interaction graphs for mean of mode II interlaminar fracture toughness.

The interaction plot for the average mode II interlaminar fracture toughness is shown in Figure 15. Since there are two factors involved, only one interaction plot is generated. The lines representing the interaction between the two factors are nearly parallel, indicating that there is no significant interaction between them. When interaction effects between factors are not significant, optimal conditions can be determined using main effects plots. Through the main effects plot, we identified the condition that maximizes the average mode II interlaminar fracture toughness. It was confirmed that the mode II interlaminar fracture toughness is maximized under the co-bonding adhesive bonding and peel ply treatment condition. This conclusion aligns with the optimal condition identified from the test results in Table 10.

To numerically verify the significance of main effects and interactions, the response factor selected was the mode II interlaminar fracture toughness, and an ANOVA was performed. Table 11 presents the results of the ANOVA for mode II interlaminar fracture toughness. From the ANOVA results, the *p*-values for both factors were found to be less than the significance level of 0.05, indicating that their relationship with the response factor is statistically significant. Therefore, it can be concluded that both factors significantly influence the mode II interlaminar fracture toughness.

### 4.2. Low-Velcocity Impact Simulation

#### 4.2.1. Validation

**Figure 16:**
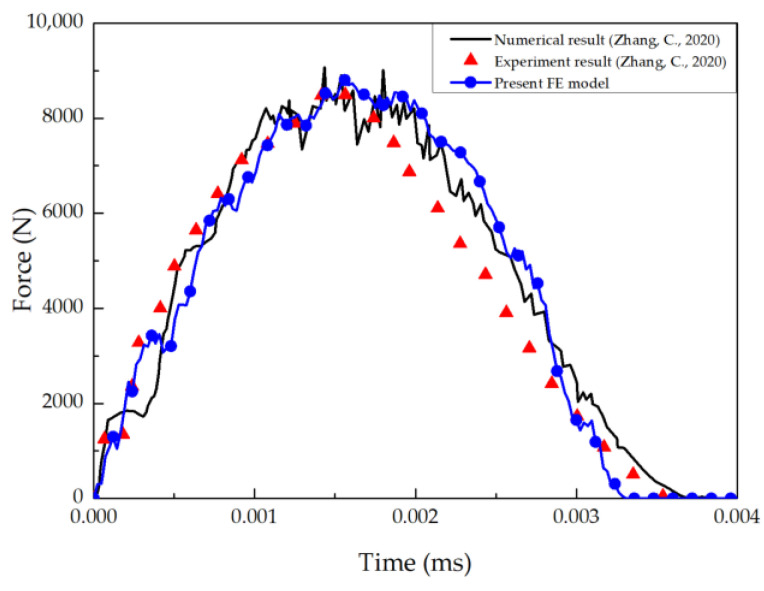
Comparison of force–time graph for verifying the low-velocity impact simulation.

To validate the low-velocity impact analysis of composite laminates using CZM, comparisons were made between the experimental results, the numerical results from the reference literature, and the analysis results obtained in the present study. A load-time graph, as shown in Figure 16, was plotted to compare the performance of the present FE model. Table 12 presents a detailed comparison of the maximum force and impact energy.

The experimental maximum force is 8498.4 N, while the numerical result from the reference is 9070.3 N. In comparison, the maximum force obtained from the present FE model was 8805.2 N. The relative errors between the present FE model and the experimental and numerical results were 3.61% and 2.92%, respectively. These results indicate good agreement between the present FE model and both the experimental and numerical results from the reference literature. Relative error for the maximum load is found to be 2.92%. Hence, the numerical conditions applied in the present FE model were validated, confirming the reliability for predicting low-velocity impact behavior. Based on the validated conditions, low-velocity impact analysis was performed.

#### 4.2.2. Simulation Incorporating Experimental Interlaminar Fracture Toughness

To investigate the influence of temperature, surface treatment techniques, and adhesion processes on interlaminar fracture toughness affecting the impact behavior of laminates, low-velocity impact analysis was conducted. Damage areas in the composite base and adhesive were identified. Specifically, no damage was observed in the composite fibers in any direction, whereas damage occurred in the composite base due to the impact. This was attributed to the impact energy not being sufficient to cause damage to the fibers in the composite. Therefore, the analysis focused on comparing and analyzing damage areas in the matrix under each analysis condition. During low-velocity impact on the composite laminate, compression stress was applied on the upper side while tensile stress was applied on the lower side. Consequently, damage in the matrix was confirmed to occur due to both tensile and compressive stress.

**Figure 17:**
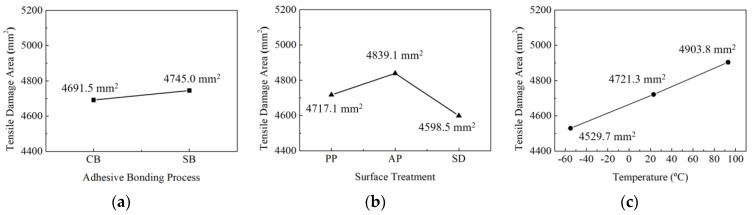
Comparison of matrix tensile damage area as experimental factors: (**a**) adhesion process; (**b**) surface treatment; (**c**) temperature.

To compare the size of tensile damage areas in the base according to different factors, averages were calculated based on the results in Table 13, and the results were represented graphically as shown in Figure 17. Regarding adhesion processes, the damage area was significantly larger with secondary adhesion (4745.0 mm^2^) compared to co-bonding adhesion (4691.5 mm^2^), as shown in Figure 17a. Surface treatment resulted in varying damage area sizes with fuse plasma treatment (4839.1 mm^2^), peel ply treatment (4717.1 mm^2^), and sanding (4598.5 mm^2^) in descending order, as shown in Figure 17b. In terms of temperature, damage area sizes were observed in the order of 93 °C (4903.8 mm^2^), 23 °C (4721.3 mm^2^), and −53 °C (4529.7 mm^2^), as shown in Figure 17c. This suggests that lower temperatures increase the Hashin damage properties of the composite, resulting in smaller areas of damage.

**Figure 18:**
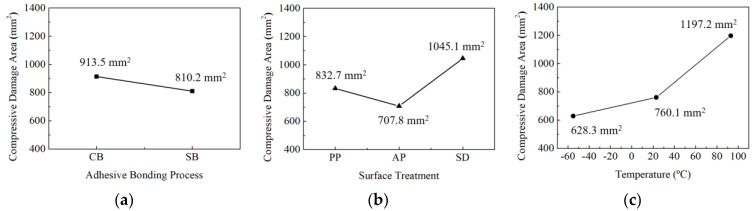
Comparison of matrix compressive damage area as experimental factors: (**a**) adhesion process; (**b**) surface treatment; (**c**) temperature.

To compare the size of compressive damage areas in the base according to different factors, averages were calculated based on the results in Table 13, and the results were represented graphically as shown in Figure 18. The damage area was significantly larger with co-bonding adhesion (913.5 mm^2^) compared to secondary adhesion (810.2 mm^2^), as shown in Figure 18a. Regarding surface treatments, the damage area sizes appeared in the order of sanding (1045.1 mm^2^), peel ply treatment (832.7 mm^2^), and fuse plasma treatment (707.8 mm^2^), as shown in Figure 18b. Concerning temperature, the damage area sizes were observed in the order of 93 °C (1197.2 mm^2^), 23 °C (760.1 mm^2^), and −53 °C (628.3 mm^2^), as shown in Figure 18c. Similar to tensile damage areas in the base, this indicates that lower temperatures increase the Hashin damage properties of the composite, resulting in smaller areas of damage.

**Figure 19:**
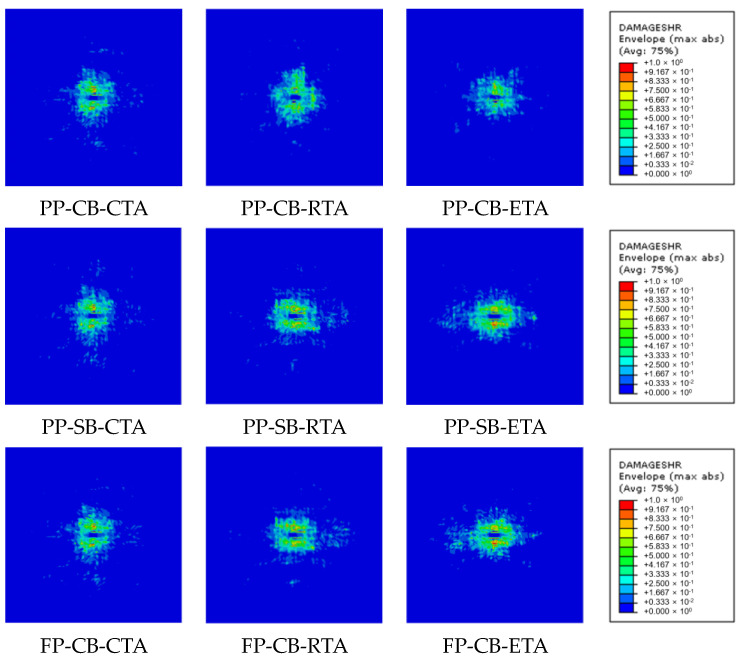

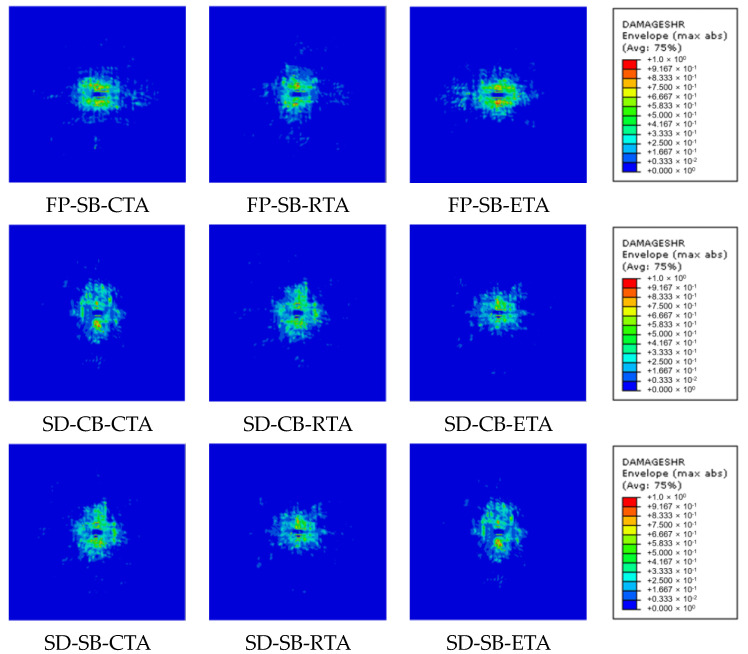
Comparison of delamination area based on composite adhesion processes and test environmental conditions.

**Figure 20:**
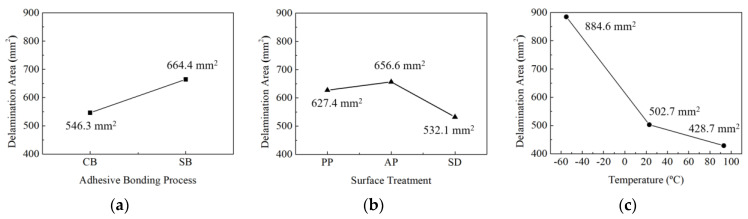
Comparison of delamination area as experimental factors: (**a**) adhesion process; (**b**) surface treatment; (**c**) temperature.

Figure 19 illustrates the interlaminar delamination areas in the adhesion joints caused by low-velocity impact. The influence of each factor was assessed through comparisons of the delamination areas. Table 14 presents the sizes of delamination areas under different conditions. Figure 20 compares the delamination areas according to each factor. It can be observed that the trend in the size of the delamination area in terms of the experimental factors shows an opposite tendency to the trend of mode I interlaminar fracture toughness shown in Figure 12. The delamination area was significantly larger with secondary adhesion (664.4 mm^2^) compared to co-bonding adhesion (546.3 mm^2^), as shown in Figure 20a. Regarding surface treatments, the delamination area sizes appeared in the order of fuse plasma treatment (656.6 mm^2^), peel ply treatment (627.4 mm^2^), and sanding (532.1 mm^2^), as shown in Figure 20b. Regarding temperature, the delamination area sizes were observed in the order of −53 °C (884.6 mm^2^), 23 °C (502.7 mm^2^), and 93 °C (428.7 mm^2^), as shown in Figure 20c. Through the analysis of the results, it was confirmed that as the mode I interlaminar fracture toughness increases, the delamination area size decreases. Therefore, it can be concluded that the combination of experimental parameters resulting in the smallest delamination area of 319.0 mm^2^ is the co-bonding process, sanding, and high temperature. Under these conditions, the mode I interlaminar fracture toughness is the highest at 1.384 kJ/m^2^.

## 5. Conclusions

Ensuring the reliable performance of composite adhesive joints under low-velocity impact is essential for maintaining the structural durability of composite materials in demanding applications. This study investigated the effects of temperature, surface treatment techniques, and bonding processes on interlaminar fracture toughness. Using a Taguchi experimental design and analysis of variance (ANOVA), the study systematically evaluated these factors and applied the experimentally derived toughness values to low-velocity impact simulations to assess delamination behavior.

We analyzed the main effects and interactions of each experimental factor (temperature, surface treatment, bonding process). In the DCB tests, all three factors showed significant effects. In the ENF tests, while surface treatment and bonding process showed significant effects, the influence of temperature was not considered due to the limitations of the testing setup. This represents a limitation of the study and highlights the need for future research to explore the effects of temperature on ENF performance. However, interactions did not significantly influence the mode I and mode II interlaminar fracture toughness values. The results of the analysis of variance confirmed that all factors were statistically significant, with *p*-values lower than the significance level of 0.05.

The low-velocity impact analysis performed on composite plates using the derived interlaminar fracture toughness showed that no damage occurred in the composite fibers, with damage only observed in the matrix and adhesion regions. At lower temperatures, the Hashin damage properties increased in the matrix, resulting in reduced damage. Conversely, in the adhesion regions, as mode I interlaminar fracture toughness increased, the size of the delamination area decreased. Notably, the combination of high temperature with co-bonding and sanding yielded the smallest delamination area of 319.0 mm^2^, demonstrating superior damage resistance of the composite structure. These findings emphasize the practical implications for automotive doors or aircraft structural joints, and the potential of processes that enhance interlaminar fracture toughness.

Future research should focus on developing methods to analyze the effects of temperature in ENF tests, particularly under extreme environmental conditions. Additionally, investigating dynamic impact scenarios and their correlation with interlaminar fracture toughness is essential to better understand composite material performance across various bonding processes. By improving understanding and control of these factors, our study contributes valuable insights into optimizing composite material performance and durability in demanding applications.

## Figures and Tables

**Table 1:** Factors and levels of DCB and ENF tests.

Test	Variables	Factors	Level		
DCB	A	Temperature	−53 °C	23 °C	93 °C
	B	Surface treatment	Peel ply	Fuse plasma	Sanding
	C	Adhesive process	Co-bonding	Secondary bonding	-
ENF	D	Surface treatment	Peel ply	Fuse plasma	Sanding
	E	Adhesive process	Co-bonding	Secondary bonding	-

**Table 2:** Intralaminar material properties of T700/M21 composites.

Mechanical Properties			Hashin Damage Variables		
Symbol	Value	Unit	Symbol	Value	Unit
*E*_11_	130	GPa	*X^t^*	2080	MPa
*E*_22_	7.7	GPa	*X^c^*	1250	MPa
*E*_33_	7.7	GPa	*Y^t^*	60	MPa
*G*_12_	4.8	GPa	*Y^c^*	140	MPa
*G*_13_	4.8	GPa	*S*	110	MPa
*G*_23_	3.8	GPa	*G_ft_*	133	N/mm
ν _12_	0.33	-	*G_fc_*	40	N/mm
ν _13_	0.33	-	*G_mt_*	0.6	N/mm
ν _23_	0.35	-	*G_mc_*	2.1	N/mm

**Table 3:** Interlaminar material properties of T700/M21 composites.

Mechanical Properties			Hashin Damage Variables		
Symbol	Value	Unit	Symbol	Value	Unit
*K_nn_*	5000	N/mm^3^	*T*_1_ *, T*_2_ *, T*_3_	30	MPa
*K_ss_*	5000	N/mm^3^	*G_1_*_c_	0.6	N/mm
*K_tt_*	5000	N/mm^3^	*G_2_*_c_ *, G_3_*_c_	2.1	N/mm

**Table 4:** Thermal material properties of MTM45/IM7 and FM309.

Material	Thermal Expansion (με/K)	Thermal Conductivity (W/m·K)
MTM45/IM7	$\alpha_{1}$ = −5.5 $\alpha_{2}$ = 28.5	0.2
FM309	5.9	0.5

**Table 5:** Mechanical properties of MTM45/IM7.

Case	Modulus (GPa)	Poisson’s Ratio
CTA	*E*_11_ = 154, *E*_22_ = *E*_33_ = 8.55 *G*_12_ = *G*_13_ = *G*_23_ = 4.36	ν_12_ = ν_13_ = ν_23_ = 0.346
RTA	*E*_11_ = 152, *E*_22_ = *E*_33_ = 7.65 *G*_12_ = *G*_13_ = *G*_23_ = 3.62	ν_12_ = ν_13_ = ν_23_ = 0.361
ETA	*E*_11_ = 147, *E*_22_ = *E*_33_ = 6.55 *G*_12_ = *G*_13_ = *G*_23_ = 4.36	ν_12_ = ν_13_ = ν_23_ = 0.373

**Table 6:** Hashin damage variables of MTM45/IM7.

Case	Hashin Damage	
	Initiation (MPa)	Evolution (N/mm)
CTA	*X^t^* = 2442, *X^c^* = 1383 *Y^t^* = 57.5, *Y^z^* = 263.8 *S^1^* = 53.4, *S^t^* = 144	*G_ft_* = 195.8, *G_fc_* = 165.6 *G_mt_* = 0.348, *G_mc_* = 0.884
RTA	*X^t^* = 2369, *X^c^* = 1226 *Y*^t^ = 52.3, *Y^z^* = 192.8 *S^1^* = 40.7, *S^t^* = 100	*G_ft_* = 195.8, *G_fc_* = 165.6 *G_mt_* = 0.348, *G_mc_* = 0.884
ETA	*X^t^* = 2453, *X^c^* = 1063 *Y^t^* = 29.6, *Y^z^* = 108.3 *S^1^* = 24.3, *S^t^* = 58.9	*G_ft_* = 195.8, *G_fc_* = 165.6 *G_mt_* = 0.348, *G_mc_* = 0.884

**Table 7:** Mechanical material properties of FM309.

No.	Traction (N/mm^3^)	QUADS Damage		Density (ton/mm^3^)
		Initiation (MPa)	Evolution (N/mm)	
1	*K_nn_*, *K_ss_*, *K_tt_* = 8000	*T_1_* = 6.7 *T_2_*, *T_3_* = 27.6	*G_1c_* = 0.635 *G_2c_, G_3c_* = 4.718	1.12 × 10^−9^
2	*K_nn_*, *K_ss_*, *K_tt_* = 4300	*T_1_* = 6.4 *T_2_*, *T_3_* = 37	*G_1c_* = 1.305 *G_2c_, G_3c_* = 4.718	
3	*K_nn_*, *K_ss_*, *K_tt_* = 4000	*T_1_* = 6.1 *T_2_*, *T_3_* = 33.7	*G_1c_* = 1.342 *G_2c_, G_3c_* = 4.718	
4	*K_nn_*, *K_ss_*, *K_tt_* = 8500	*T_1_* = 6.7 *T_2_*, *T_3_* = 27.6	*G_1c_* = 0.594 *G_2c_, G_3c_* = 4.614	
5	*K_nn_*, *K_ss_*, *K_tt_* = 4500	*T_1_* = 6.4 *T_2_*, *T_3_* = 37	*G_1c_* = 1.247 *G_2c_, G_3c_* = 4.614	
6	*K_nn_*, *K_ss_*, *K_tt_* = 3000	*T_1_* = 6.1 *T_2_*, *T_3_* = 33.7	*G_1c_* = 1.345 *G_2c_, G_3c_* = 4.614	
7	*K_nn_*, *K_ss_*, *K_tt_* = 6800	*T_1_* = 6.7 *T_2_*, *T_3_* = 27.6	*G_1c_* = 0.668 *G_2c_, G_3c_* = 4.437	
8	*K_nn_*, *K_ss_*, *K_tt_* = 4000	*T_1_* = 6.4 *T_2_*, *T_3_* = 37	*G_1c_* = 1.310 *G_2c_, G_3c_* = 4.437	
9	*K_nn_*, *K_ss_*, *K_tt_* = 3800	*T_1_* = 6.1 *T_2_*, *T_3_* = 33.7	*G_1c_* = 1.384 *G_2c_, G_3c_* = 4.437	
10	*K_nn_*, *K_ss_*, *K_tt_* = 8500	*T_1_* = 6.7 *T_2_*, *T_3_* = 27.6	*G_1c_* = 0.509 *G_2c_, G_3c_* = 4.133	
11	*K_nn_*, *K_ss_*, *K_tt_* = 5000	*T_1_* = 6.4 T_2_, T_3_ = 37	*G_1c_* = 0.886 *G_2c_, G_3c_* = 4.133	
12	*K_nn_*, *K_ss_*, *K_tt_* = 4000	*T_1_* = 6.1 *T_2_*, *T_3_* = 33.7	*G_1c_* = 0.901 *G_2c_, G_3c_* = 4.133	
13	*K_nn_*, *K_ss_*, *K_tt_* = 9000	*T_1_* = 6.7 *T_2_*, *T_3_* = 27.6	*G_1c_* = 0.352 *G_2c_, G_3c_* = 3.931	
14	*K_nn_*, *K_ss_*, *K_tt_* = 5000	*T_1_* = 6.4 *T_2_*, *T_3_* = 37	*G_1c_* = 0.308 *G_2c_, G_3c_* = 3.931	
15	*K_nn_*, *K_ss_*, *K_tt_* = 4000	*T_1_* = 6.1 *T_2_*, *T_3_* = 33.7	*G_1c_* = 0.873 *G_2c_, G_3c_* = 3.931	
16	*K_nn_*, *K_ss_*, *K_tt_* = 7000	*T_1_* = 6.7 *T_2_*, *T_3_* = 27.6	*G_1c_* = 0.568 *G_2c_, G_3c_* = 3.789	
17	*K_nn_*, *K_ss_*, *K_tt_* = 5000	*T_1_* = 6.4 *T_2_*, *T_3_* = 37	*G_1c_* = 1.129 *G_2c_, G_3c_* = 3.789	
18	*K_nn_*, *K_ss_*, *K_tt_* = 5000	*T_1_* = 6.1 *T_2_*, *T_3_* = 33.7	*G_1c_* = 1.164 *G_2c_, G_3c_* = 3.789	

**Table 8:** Results of mode I interlaminar fracture toughness tests.

No.	Adhesion Process	Surface Treatment	Temp. (°C)	*G_Ic_* (kJ/m^2^)
1	CB	PP	−53	0.572
2	CB	PP	23	1.436
3	CB	PP	93	1.543
4	CB	FP	−53	0.535
5	CB	FP	23	1.060
6	CB	FP	93	1.210
7	CB	SD	−53	0.534
8	CB	SD	23	1.441
9	CB	SD	93	1.660
10	SB	PP	−53	0.504
11	SB	PP	23	0.930
12	SB	PP	93	1.108
13	SB	FP	−53	0.422
14	SB	FP	23	0.550
15	SB	FP	93	1.091
16	SB	SD	−53	0.625
17	SB	SD	23	1.242
18	SB	SD	93	1.280

**Table 9:** Statistical analysis of mode I test results.

Category	*DF*	*SS*	*MS*	*F*	*p*
Adhesion process	1	0.48213	0.48213	27.48	0
Surface treatment	2	0.14379	0.07189	4.1	0.044
Temperature	2	1.29316	0.64658	36.86	0
Error	12	0.21052	0.01754		
Statics	17	2.12959			

**Table 10:** Results of mode II interlaminar fracture toughness tests.

No.	Adhesion Process	Surface Treatment	*G_IIc_* (kJ/m^2^)
1	CB	PP	4.624
2	CB	FP	4.522
3	CB	SD	4.348
4	SB	PP	3.720
5	SB	FP	3.538
6	SB	SD	3.410

**Table 11:** Statistics analysis of mode II test results.

Category	*DF*	*SS*	*MS*	*F*	P
Adhesion process	1	0.61197	0.61197	498.43	0.002
Surface treatment	2	0.09773	0.04886	39.8	0.025
Error	12	0.00246	0.00123		
Statics	17	0.71215			

**Table 12:** Validation of low-velocity impact simulation.

Category	Force (N)	Impact Energy (J)	Error (%)
Numerical result [30]	9070.3	25.0	2.92
Experiment result [30]	8498.4	25.0	3.61
Present FE model	8805.2	25.0	-

**Table 13:** Damage area of composite matrix under tensile and compressive conditions.

Case	Tensile Damage Area of Composite Matrix (mm^2^)	Compressive Damage Area of Composite Matrix (mm^2^)
PP-CB-CTA	4590.2	543.1
PP-CB-RTA	4573.7	920.9
PP-CB-ETA	4721.7	1473.4
FP-CB-CTA	4576.4	526.6
FP-CB-RTA	4847.2	689.9
FP-CB-ETA	5166.5	962.7
SD-CB-CTA	4349.6	832.5
SD-CB-RTA	4676.6	810.3
SD-CB-ETA	4721.3	1462.1
PP-SB-CTA	4587.1	523.3
PP-SB-RTA	4791.5	625.2
PP-SB-ETA	5038.5	910.1
FP-SB-CTA	4601.7	527.8
FP-SB-RTA	4805.2	629.9
FP-SB-ETA	5037.7	909.9
SD-SB-CTA	4473.3	816.5
SD-SB-RTA	4633.3	884.2
SD-SB-ETA	4737.2	4737.2

**Table 14:** Delamination area of composite plates as adhesion conditions.

Case	Delamination Area (mm^2^)
PP-CB-CTA	899.3
PP-CB-RTA	429.1
PP-CB-ETA	340.8
FP-CB-CTA	966.7
FP-CB-RTA	471.7
FP-CB-ETA	366.0
SD-CB-CTA	708.8
SD-CB-RTA	414.9
SD-CB-ETA	319.0
PP-SB-CTA	965.5
PP-SB-RTA	583.0
PP-SB-ETA	546.5
FP-SB-CTA	1005.4
FP-SB-RTA	579.4
FP-SB-ETA	550.4
SD-SB-CTA	761.8
SD-SB-RTA	538.3
SD-SB-ETA	449.7

## Data Availability

The original contributions presented in this study are included in the article. Further inquiries can be directed to the corresponding author.
